# Health inequity in access to bariatric surgery: a protocol for a systematic review

**DOI:** 10.1186/2046-4053-3-15

**Published:** 2014-02-21

**Authors:** Timothy D Jackson, Rujun Zhang, Dresden Glockler, Jason Pennington, Jacinta I Reddigan, Ori D Rotstein, Janet Smylie, Laure Perrier, Lesley Gotlib Conn

**Affiliations:** 1 Division of General Surgery, University Health Network, 399 Bathurst Street, Toronto, ON M5T 2S8, Canada; 2 University of Ottawa, 75 Laurier Avenue East, Ottawa, ON K1N 6 N5, Canada; 3 University of Western Ontario, 1151 Richmond St, London, ON N6A 3 K7, Canada; 4 Division of General Surgery, The Scarborough Hospital, 3050 Lawrence Avenue East, Scarborough, ON M1P 2 V5, Canada; 5 Department of Surgery, St. Michael’s Hospital, 30 Bond Street, Toronto, ON M5B 1 W8, Canada; 6 Centre for Research on Inner City Health, Keenan Research Centre of the Li Ka Shing Knowledge Institute, St. Michael’s Hospital, 30 Bond Street, Toronto, ON M5B 1 W8, Canada; 7 Knowledge Translation Program, Li Ka Shing Knowledge Institute, St. Michael’s Hospital, 30 Bond Street, Toronto, ON M5B 1 W8, Canada; 8 Sunnybrook Research Institute, 2075 Bayview Avenue, K3W-27, Toronto, ON M4N 3 M5, Canada

**Keywords:** Bariatric surgery, Sociodemographic disparities, Obesity, Systematic review

## Abstract

**Background:**

Bariatric surgery is the only weight-loss treatment available that results in both sustained weight loss and improvements of obesity-related comorbidities. Individuals who meet the eligibility criteria for bariatric surgery are generally older, come from racial or ethnic minorities, are economically disadvantaged, and have low levels of education. However, the population who actually receives bariatric surgery does not reflect the individuals who need it the most. The objective is to conduct a systematic review of the literature exploring the inequities to the access of bariatric surgery.

**Methods/Design:**

EMBASE and Medline databases will be searched for observational studies that compared at least one of the PROGRESS-PLUS sociodemographic characteristics of patients eligible for bariatric surgery to those who actually received the procedure. Articles published in the year 1980 to present with no language restrictions will be included. For inclusion, studies must only include adults (≥18 years old) who meet National Institutes of Health (NIH) eligibility criteria for bariatric surgery defined as having either (1) a body mass index (BMI) of 40 kg/m^2^ or greater; or (2) BMI of 35 kg/m^2^ or greater with significant weight-related comorbidities. Eligible interventions will include malabsorptive, restrictive, and mixed bariatric procedures.

**Discussion:**

There appears to be inequities in access to bariatric surgery. In order to resolve the health inequity in the treatment of obesity, a synthesis of the literature is needed to explore and identify barriers to accessing bariatric surgery. It is anticipated that the results from this systematic review will have important implications for advancing solutions to minimize inequities in the utilization of bariatric surgery.

http://www.crd.york.ac.uk/PROSPERO/display_record.asp?ID=CRD42013004920.

## Background

Obesity is the leading cause of preventable death in the developed world [1,2]. Since the 1970’s, the prevalence of obesity has increased dramatically. Recent estimates indicate that 24% of Canadian adults and 34% of American adults are obese, defined as having a Body Mass Index (BMI) ≥30 kg/m^2^[3]. The prevalence of morbid obesity, defined as having a BMI ≥40 kg/m^2^, has increased by 400% in the past two decades [4,5]. The reduced quality of life and the life expectancy associated with obesity due to obesity-related comorbidities [6,7], such as diabetes and hypertension, are even more pronounced among those who are classified as morbidly obese [8]. The current obesity epidemic reflects the limited benefits of medically supervised weight-loss interventions to sustain weight loss [9,10].

Bariatric surgery has been documented as being the only treatment available that results in sustained weight loss, leading to significant improvement in obesity-related comorbidities [11]. Indeed, bariatric surgical procedures significantly decrease overall mortality in those patients who received such procedures compared to controls [12]. The current indications for bariatric surgery as determined by the National Institutes of Health (NIH) criteria [13] include patients with a BMI of 40 kg/m^2^ or greater, or a BMI of 35 kg/m^2^ or greater with significant weight-related comorbidities. When bariatric surgery is delivered in the appropriate model of care, these procedures are associated with low morbidity and mortality [14-17] and have been proven to be cost effective [18-20].

Despite the evidence supporting the safety, clinical benefits and cost effectiveness of bariatric surgery, uptake of these procedures in eligible patients remains low [21]. Health inequity, defined as unfair inequalities in population groups that lead to unequal chances to access health care services [22], may be to blame. Studies have documented significant disparities between the general morbidly obese population and the subset that have access to and/or receive bariatric surgical procedures [23-25]. Compared to the general population, individuals who fulfill the NIH criteria, and therefore are candidates for bariatric surgery, are often older, come from racial or ethnic minorities, are economically disadvantaged, and have low levels of education [23,26]. However, it is this subset of the population that is least likely to have access to bariatric surgery [25].

In an effort to create equity in the access to bariatric surgery for the treatment of obesity, a clear understanding of the apparent disparities is required. The acronym PROGRESS-Plus describes the sociodemographic factors across which disadvantage may exist: **P**lace of residence; **R**ace/ethnicity/culture; **O**ccupation; **G**ender/sex; **R**eligion; **E**ducation; **S**ocioeconomic status; **S**ocial capital; **Plus** - additional factors (for example, age) [27,28]. This protocol describes a systematic review that aims to identify the PROGRESS-Plus factors that differ between morbidly obese patients who are eligible for weight reduction surgery and those who actually receive this operation.

## Methods

### Eligibility

#### Types of studies

Retrospective and prospective cohort studies, case series, case control, and cross-sectional survey studies will be included, irrespective of the blinding employed (for example, single-blinded or open). To be included, studies must compare study participants on at least one of the PROGRESS-Plus factors.

#### Types of participants

Adult patients over the age of 18 meeting the NIH criteria for bariatric surgery will be included, irrespective of geographical location. The NIH criteria for bariatric surgery include having: (1) a BMI ≥40 kg/m^2^ or (2) a BMI ≥35 kg/m^2^ with at least one significant weight-related comorbidity [13].

#### Types of intervention

The intervention must be a bariatric surgery that may include the following procedure types: Roux-en-Y gastric bypass, sleeve gastrectomy, adjustable gastric band, vertical banded gastroplasty, jejunoileal bypass, biliopancreatic diversion, duodenal switch, mini-gastric bypass, loop gastric bypass, gastric placation, gastric balloon, or scopinaro procedure. Both open and laparoscopic approaches will be considered.

Interventions may be either universally implemented or targeted to a specific risk group. The control group must be assigned to receive no intervention, standard care or assigned to a wait-list group.

### Outcome

#### Main outcome

The outcome of interest is the utilization of bariatric surgery. Two groups have been *a priori* defined as (1) patients who are eligible for bariatric surgery and receive the procedure, and (2) those who are eligible for bariatric surgery but do not receive the procedure.

#### Sociodemographic factors

The PROGRESS-PLUS sociodemographic factors will be used to explore the factors associated with bariatric surgery delivery. **P**lace of residence will be categorized by the geographical location of residence classified as urban, suburban and rural. **R**ace/ethnicity/culture will be defined by ethnicity with the following categories: British; Eastern European, Western European, Asian, South Asian, Black, Hispanic, Aboriginal, and Other. **O**ccupation will be categorized as being professional, skilled, unskilled and unemployed. **G**ender/sex will be categorized as being male or female. **R**eligion will be categorized as identifying with Christianity/Catholicism, Judaism, Islam and Other. **E**ducation will be categorized by the highest level of education attained (graduate; post-secondary, secondary and primary). **S**ocioeconomic status will be documented as median and interquartile range (IQR) household income and will be categorized into the following income categories: <$50,000, between 50,000 and $99,000, or ≥ $100,000. **S**ocial capital will be defined by family support as being full support, some support or no support. **Plus factors** will include age and health insurance. Age will be documented as mean interquartile range and categorized into the following age categories: 18 to 19 years, 20 to 39 years, 40 to 59 years, and 60+ years. Health insurance will be defined by the type of insurance, classified as being universal, private or none.

### Search strategy

Studies will be indentified through the bibliographic databases of EMBASE, Medline (See Appendix 1 in Additional file 1), and hand searching of journals, meeting abstracts, technical or research reports, monographs, doctoral dissertations, bibliographies of retrieved papers, and relevant web sites. In addition, unpublished studies and gray literature will be sought through internet searches with specific websites targeted, including:

1. Statistics Canada: http://www.statcan.gc.ca/start-debut-eng.html

2. Royal College of Physicians and Surgeons of Canada: http://www.royalcollege.ca/

3. Institute for Clinical Evaluative Sciences: http://www.ices.on.ca/index.html

4. Canadian Institute for Health Information: http://www.cihi.ca

5. American College of Surgeons: http://www.facs.org/

6. National Centre for Health Statistics: http://www.cdc.gov/nchs/

7. National Institutes of Health: http://www.nih.gov/

8. Agency for Healthcare Research and Quality: http://www.ahrq.gov/

9. Royal College of Surgeons of England: http://www.rcseng.ac.uk/

10. Health and Social Care Information Centre: http://www.hscic.gov.uk/

11. European Institute for Health Records: http://www.eurorec.org/

12. Royal Australasian College of Surgeons: http://www.surgeons.org/

13. Australian Institute for Health and Welfare: http://www.aihw.gov.au/

The search strategies will be developed by an experienced librarian and peer reviewed using Peer Review of Electronic Search Strategy (PRESS) [29]. The final search strategy will combine medical subject headings (MeSH terms) and appropriate wildcards. No publication language limit will be set during the database searches. A lower date limit of 1980 will be set as the availability of bariatric surgery prior to 1980 was not widespread.

### Study selection

Two research assistants will review the title, abstraction or description of all trials identified by the literature search. Those studies that aim to explore bariatric surgery among individuals over the age of 18 will be selected for full-text review to determine if they meet inclusion criteria. Any discrepancy will be resolved by a third person.

### Data extraction

Two researchers will independently extract data from included studies on a prepared data collection form. The data abstraction form will be pilot tested on a random sample of studies to ensure high inter-rater agreement between reviewers. Extracted data will include: study characteristics; primary outcome results; patient risk factors, including BMI, smoking status, mental health status, quality of life, and physical activity level; and details of the surgical intervention. The presence or absence of the following comorbidities will be documented: hypertension, dyslipidemia, diabetes, coronary artery disease, cerebrovascular disease, depression, hypothyroidism, sleep apnea, gastroesophageal reflux, osteoarthrisits, and cholelithiasis.

### Quality assessment

Two researchers will independently assess the quality of all studies included for review. Any discrepancy will be resolved by a third reviewer. To quantify the degree of bias in the included studies, the Newcastle-Ottawa Scale (NOS) will be used [30]. The NOS was developed for quality assessment of observational epidemiological studies (http://www.ohri.ca/programs/clinical_epidemiology/oxford.asp). The NOS has three categories within which risk of study bias can be determined: selection, comparability and outcome. In assessing risk of bias in cohort studies, the NOS awards a ranking for the selection of the cohort, comparability of the cohort and for the assessment of outcomes. In assessing risk of bias in case–control studies, the NOS awards a ranking for the selection of cases and controls, in the comparability of cases and controls and for the ascertainment of the exposure.

### Data synthesis and analysis

Health inequities in accessing bariatric surgery will be explored using two of the following methods: first, data permitting, metaregression via a multivariate logistic regression using study-level data will be used to explore the PROGRESS-PLUS factors associated with the utilization of bariatric surgery (yes/no). This analysis will allow for the assessment of the impact of any individual covariates on utilization rates, as well as of a potential effect modifier. In addition, differences in PROGRESS-PLUS factors between the surgery and no surgery group within individual studies will be explored. The proportion of study participants categorized within each PROGRESS-PLUS category will be summarized as a percentage with a corresponding 95% confidence interval (CI) for dichotomous and categorical variables, and as median and interquartile range (IQR) for continuous variables. Second, differences in PROGRESS-PLUS factors between groups will be compared using χ^2^ test or Fisher’s exact test for categorical variables and the Wilcoxon-Mann–Whitney test for continuous variables. Statistical tests will be carried out as 2-tailed tests at α = 0.05. The DerSimonian and Laird method will be used to test heterogeneity of effect sizes between studies. Heterogeneity will be assumed at *P* <0.05 and I^2^ ≥25%. Data will be analyzed with SAS (version 9.1; SAS Institute Inc. Cary, NC, USA).

Irrespective of the presence or absence of heterogeneity the following subgroup analyses on the main outcome will be performed to explore possible effect modifications:

1. Participants characteristics

2. Intervention type; and

3. Study quality

## Discussion

While bariatric surgery has been demonstrated to be an effective treatment for obesity, it appears that access to, and uptake of bariatric surgery does not uniformly match with populations with high rates of obesity and obesity-related diseases [23-25]. Factors such as ethnicity, age, sex, socioeconomic status, geographic location and others appear to play an important role in determining access to care. In order to address barriers and move toward health equity in the treatment of obesity, a synthesis of the literature is needed to fully explore and identify the gaps in our understanding of access to bariatric surgery. We will disseminate the results of this review in an open access scientific journal and will present results at scientific conferences. We expect our results will have important implications for the delivery of bariatric surgery by providing some leads to the barriers to accessing these operations. Findings of inequities in the access to bariatric surgery will be used to inform the design of qualitative research which will provide insight into what drives the identified factors to act as barriers and help prioritize solutions to bridge the care gap.

## Abbreviations

BMI: body mass index; NIH: National Institutes of Health; MeSH: Medical Subject Headings; NOS: Newcastle-Ottawa Scale.

## Competing interests

The authors declare that they have no competing interests.

## Authors’ contributions

TJ collaborated in the design of the study and drafted the protocol. ODR conceived the study, collaborated in the design of the study, and edited the protocol. RZ collaborated in the design of the study and edited the protocol. DG collaborated in the design of the study and edited the protocol. JP collaborated in the design of the study and edited the protocol. JIR collaborated in the design of the study and edited the protocol. JS collaborated in the design of the study and edited the protocol. LP developed the search strategy and edited the protocol. LGC collaborated in the design of the study and edited the protocol. All authors read and approved the final manuscript.

## Supplementary Material

Additional file 1: Appendix I Literature Search Strategy. Click here for file
