# Supplementary material for: Health inequity in access to bariatric surgery: a protocol for a systematic review
Source: Syst Rev. 2014 Feb 21;3:15. doi: 10.1186/2046-4053-3-15 (PMC3936930; doi:10.1186/2046-4053-3-15)
Supplement: Additional file 1: Appendix I — Literature Search Strategy. [file 2046-4053-3-15-S1.doc]

**Appendix I - Literature Search Strategy**

Database: Ovid MEDLINE (R) Ovid MEDLINE (R) In-Process & Other Non-Indexed Citations

Search Strategy:

| 1. | exp American Native Continental Ancestry Group/ [ socially disadvantaged ] |
| --- | --- |
| 2. | exp African Continental Ancestry Group/ |
| 3. | Ethnic Groups/ |
| 4. | Health Services, Indigenous/ |
| 5. | exp Homeless Persons/ |
| 6. | Medical Indigency/ |
| 7. | Oceanic Ancestry Group/ |
| 8. | exp Public Assistance/ |
| 9. | exp Poverty/ |
| 10. | exp Residence Characteristics/ |
| 11. | Socioeconomic Factors/ |
| 12. | exp Social Welfare/ |
| 13. | exp Social Security/ |
| 14. | “Transients and Migrants”/ |
| 15. | Vulnerable Populations/ |
| 16. | aborig$.tw. |
| 17. | (alaska adj native$).tw. |
| 18. | (american adj indian$).tw. |
| 19. | african$.tw. |
| 20. | blacks.tw. |
| 21. | caribbean$.tw. |
| 22. | disadvantag$.tw. |
| 23. | dispossess$.tw. |
| 24. | destitut$.tw. |
| 25. | eskimo$.tw. |
| 26. | emigrant$.tw. |
| 27. | (first adj nation?).tw. |
| 28. | homeless$.tw. |
| 29. | immigrant$.tw. |
| 30. | impover$.tw. |
| 31. | indigen$.tw. |
| 32. | itinerant$.tw. |
| 33. | inuit$.tw. |
| 34. | inuk.tw. |
| 35. | (low adj income$).tw. |
| 36. | metis.tw. |
| 37. | maor$.tw. |
| 38. | minorit$.tw. |
| 39. | migrant$.tw. |
| 40. | (native adj american$).tw. |
| 41. | (native adj hawaiin$).tw. |
| 42. | negro$.tw. |
| 43. | poverty$.tw. |
| 44. | poor$.tw. |
| 45. | remote$.tw. |
| 46. | (residenc$ adj characteristic$).tw. |
| 47. | tribe$.tw. |
| 48. | tribal$.tw. |
| 49. | transient$.tw. |
| 50. | “torres strait islander$”.tw. |
| 51. | underprivileg$.tw. |
| 52. | under-privileg$.tw. |
| 53. | (vulnerabl$ adj communit$).tw. |
| 54. | (vulnerabl$ adj group$).tw. |
| 55. | (vulnerabl$ adj individual$).tw. |
| 56. | (vulnerabl$ adj famil$).tw. |
| 57. | (vulnerabl$ adj population$).tw. |
| 58. | (vulnerabl$ adj people$).tw. |
| 59. | (vulnerabl$ adj person$).tw. |
| 60. | (isolat$ adj communit$).tw. |
| 61. | (isolat$ adj group$).tw. |
| 62. | (isolat$ adj individual$).tw. |
| 63. | (isolat$ adj famil$).tw. |
| 64. | (isolat$ adj population$).tw. |
| 65. | (isolat$ adj people$).tw. |
| 66. | (isolat$ adj person$).tw. |
| 67. | or/1-66 |
| 68. | exp Gastroenterostomy/ [ MeSH previous indexing 1984-2005 ] |
| 69. | limit 68 to yr = “1984 - 2005” |
| 70. | exp Bariatric Surgery/ [ bariatric surgery ] |
| 71. | (gastric adj bypass$).tw. |
| 72. | gastroplast$.tw. |
| 73. | gastrectom$.tw. |
| 74. | (duodenal adj switch).tw. |
| 75. | (jejunoileal adj bypass$).tw. |
| 76. | lipectom$.tw. |
| 77. | (gastric adj band$).tw. |
| 78. | (gastric adj placation).tw. |
| 79. | (gastric adj balloon$).tw. |
| 80. | bariatric$.tw. |
| 81. | (lap adj band$).tw. |
| 82. | (sleeve adj gastrect$).tw. |
| 83. | (biliopancreatic adj diver$).tw. |
| 84. | (bilio-pancreatic adj diver$).tw. |
| 85. | Obesity/su [Surgery] |
| 86. | (weight-loss adj surg$).tw. |
| 87. | (obes$ adj surg$).tw. |
| 88. | roux-en-y.tw. |
| 89. | (scopinaro adj procedure$).tw. |
| 90. | or/69-89 |
| 91. | 67 and 90 |
| 92. | exp Animals/ not (exp Animals/ and Humans/) [ removing animal studies ] |
| 93. | 91 not 92 |
